# Decision curve analysis revisited: overall net benefit, relationships to ROC curve analysis, and application to case-control studies

**DOI:** 10.1186/1472-6947-11-45

**Published:** 2011-06-22

**Authors:** Valentin Rousson, Thomas Zumbrunn

**Affiliations:** 1 University of Lausanne, Institute for Social and Preventive Medicine, Statistical Unit, Route de la Corniche 2, CH-1066 Epalinges, Switzerland; 2 University Hospital Basel, Clinical Trial Unit, Schanzenstrasse 55, CH-4031 Basel, Switzerland

## Abstract

**Background:**

Decision curve analysis has been introduced as a method to evaluate prediction models in terms of their clinical consequences if used for a binary classification of subjects into a group who should and into a group who should not be treated. The key concept for this type of evaluation is the "net benefit", a concept borrowed from utility theory.

**Methods:**

We recall the foundations of decision curve analysis and discuss some new aspects. First, we stress the formal distinction between the net benefit for the treated and for the untreated and define the concept of the "overall net benefit". Next, we revisit the important distinction between the concept of accuracy, as typically assessed using the Youden index and a receiver operating characteristic (ROC) analysis, and the concept of utility of a prediction model, as assessed using decision curve analysis. Finally, we provide an explicit implementation of decision curve analysis to be applied in the context of case-control studies.

**Results:**

We show that the overall net benefit, which combines the net benefit for the treated and the untreated, is a natural alternative to the benefit achieved by a model, being invariant with respect to the coding of the outcome, and conveying a more comprehensive picture of the situation. Further, within the framework of decision curve analysis, we illustrate the important difference between the accuracy and the utility of a model, demonstrating how poor an accurate model may be in terms of its net benefit. Eventually, we expose that the application of decision curve analysis to case-control studies, where an accurate estimate of the true prevalence of a disease cannot be obtained from the data, is achieved with a few modifications to the original calculation procedure.

**Conclusions:**

We present several interrelated extensions to decision curve analysis that will both facilitate its interpretation and broaden its potential area of application.

## Background

The decision to administer or not to administer a treatment against some disease is frequently based on an estimated probability *p_i _*that an individual *i *has the disease (or – in a prognostic setting – will develop the disease), typically obtained using a prediction model in the broadest sense. A treatment is then administered if *p_i _*is high enough, whereas no treatment is administered if *p_i _*is too low. To judge whether *p_i _*is high enough, one should weigh the profit *P *obtained by treating an individual with the disease and the loss *L *caused by treating an individual without the disease. The rationale is to treat individual *i *if and only if *p_i_P *> (1 - *p_i_*)*L*, i.e. if the expected profit is higher than the expected loss. Equivalently, the treatment is administered if and only if *p_i _*>*p_t_*, where *p_t _*is some threshold probability defined by *p_t_*/(1 - *p_t_*) = *L*/*P*, that is, *p_t _*= *L*/(*L *+ *P*). The threshold probability *p_t_*, and hence the decision to opt or not to opt for the treatment, is thus a one-to-one function of the ratio *L*/*P *which is informative of how a clinician or a patient weighs the relative harms of false positive and false negative results. This quantity is typically subjective and will vary from clinician to clinician and from patient to patient. In what follows, we just assume that 0 <*p_t _*< 1. Decision curve analysis consists of showing graphically the so-called "net benefit" obtained by applying the strategy of treating an individual if and only if *p_i _*>*p_t _*in function of the threshold probability *p_t _*[1]. It facilitates the comparison among alternative prediction models used to calculate *p_i_*. As a consequence, it may facilitate the decision of which of several prediction models to select, typically as a result of a clinician or a patient favouring the model with the highest net benefit at their personally determined threshold probability.

Two extreme examples of prediction models are a model for which *p_i _*= 1 for all individuals (with the consequence of all of them being treated, whatever the threshold probability), and a model for which *p_i _*= 0 for all individuals (with the consequence of none of them being treated, whatever the threshold probability). On the other hand, a model achieving a perfect prediction would provide *p_i _*= 1 for individuals with the disease and *p_i _*= 0 for individuals without the disease.

Interestingly, a binary diagnostic or prognostic test with only two possible results (positive or negative), where an individual receives the treatment if and only if the diagnostic or prognostic test is positive (an individual with a negative diagnostic or prognostic test being not treated), could be seen as a special case of a prediction model by letting *p_i _*= 1 if the test is positive for individual *i *(such that *p_i _*>*p_t_*, whatever the threshold *p_t_*), and letting *p_i _*= 0 if the test is negative (such that *p_i _*<*p_t_*, whatever the threshold *p_t_*). Thus, decision curve analysis allows the comparison of binary diagnostic or prognostic tests with other prediction models (see below for an example).

**Figure 1:**
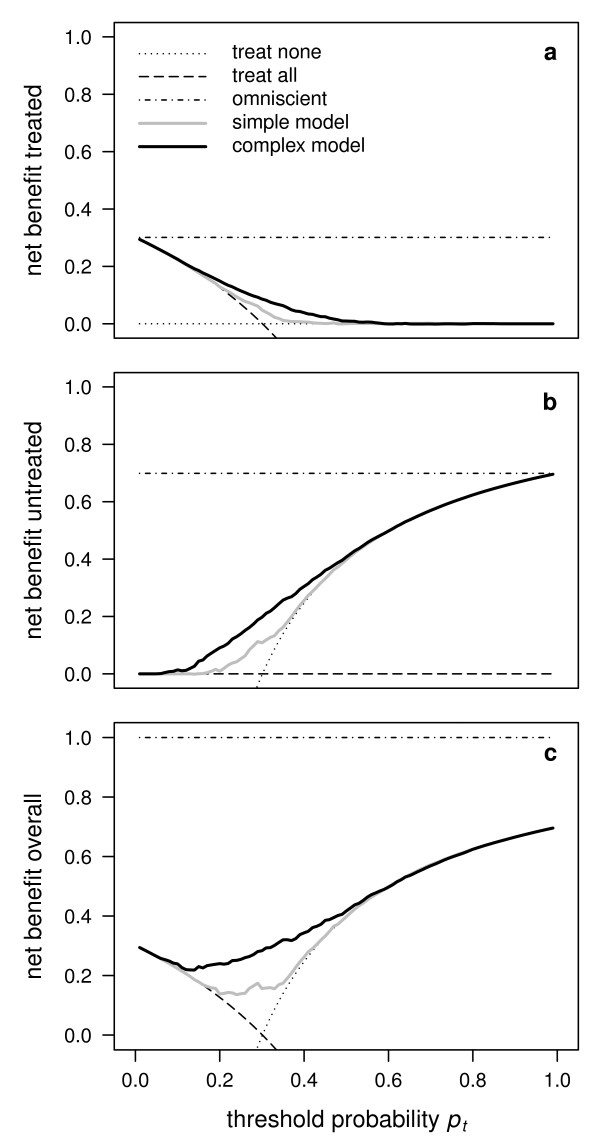
**Illustration of the net benefit for the treated, for the untreated and overall**. Decision curves based on the net benefit for the treated (a), the net benefit for the untreated (b) and the overall net benefit (c) for a simple example model and a complex example model, accompanied by the reference strategies of treating none or treating all and a hypothetical perfect prediction model ("omniscient"). Note that the addition of the decision curves for the treated (a) and the untreated (b) results in the decision curves in (c).

Consider now a random sample of the population (e.g. the sample used to calculate the *p_i_*). Let *a *be the proportion of individuals with *p_i _*>*p_t _*and with the disease, let *b *be the proportion of individuals with *p_i _*>*p_t _*and without the disease, let *c *be the proportion of individuals with *p_i _*<*p_t _*and with the disease, and let *d *be the proportion of individuals with *p_i _*<*p_t _*and without the disease (to slightly simplify our exposition, we shall assume that an equality *p_i _*= *p_t _*is not possible, such that *a *+ *b *+ *c *+ *d *= 1). Thus, *a*, *b*, *c *and *d *are (estimates of) the proportions of true positive, false positive, false negative, and true negative results, respectively, and are dependent both on the threshold *p_t _*and on the model used to calculate the *p_i_*. The prevalence of the disease is (estimated by) *π *= *a *+ *c*. Note that our use of the four letters *a*, *b*, *c *and *d *corresponds to the proportions of the four possible results of binary classification and that we thereby adopt a more commonly accepted notation (see e.g. [2]) than Vickers et al. [1] (see Figure 1 therein), who used the same letters to denote utilities of the four possible results.

Vickers et al. [1] proposed to use the so-called net benefit for the evaluation of prediction models, and to calculate it, they proceed as follows. Focusing on the individuals who receive the treatment, i.e. for whom *p_i _*>*p_t_*, the expected profit will be *aP *and the expected loss will be *bL*, the resulting benefit being *U_treated _*= *aP *- *bL*. This definition of the benefit corresponds to the "average profit per prediction" or "utility of the method" introduced by Peirce [3]. In many situations, determining actual values of *P *and *L *is not an obvious task. In decision curve analysis, it suffices to have an idea of their ratio *L*/*P *(or equivalently, of *p_t_*). In order to make the benefit dependent only on the ratio *L*/*P*, i.e. not on the actual values of *P *and *L*, Vickers et al. [1] divided the benefit by *P*, yielding the so-called net benefit, *u_treated _*= *a *- *bL*/*P*, or, expressed as a function of *p_t_*,(1)

In the extreme case where none of the individuals is treated, one has *a *= *b *= 0 and *u_treated _*= 0, which is constant whatever the threshold probability *p_t_*. In the other extreme case where all individuals are treated, one has *a *= *π*, *b *= 1 - *π *and *u_treated _*= *π *- (1 - *π*)*p_t_*/(1 - *p_t_*). This is a decreasing function of the threshold probability, ranging from the prevalence down to negative infinity. For a model achieving a perfect prediction, one has *b *= *c *= 0 and *u_treated _*= *π*, which is again constant whatever the threshold probability. For many prediction models, the empirical finding is that the net benefit for the treated will be a roughly decreasing function of the threshold probability, approximately ranging from the prevalence down to zero. 

Interpretation of the value of the net benefit is not quite straightforward. It is a difference between two complementary proportions summing up to *π*, those profiting from obtaining the treatment (since they really have the disease) minus those loosing by obtaining the treatment (since they do not have the disease and will only suffer from the treatment), the latter weighted by the ratio *L*/*P*. The net benefit is thus equal to zero if the loss compensates the profit and can even be negative if the loss surpasses the profit. The maximum possible value of the net benefit is *π*, the prevalence of the disease, which is achieved only by a perfect prediction model.

## Application to an example

To illustrate the application of decision curve analysis, we make use of a data set which is available on the accompanying website of a text book by Dupont [4]: http://biostat.mc.vanderbilt.edu/dupontwd/wddtext/data/2.20.Framingham.csv. It is a subset of the 40-year follow-up data set of the Framingham Heart Study (made available by Levy [5]). We define death due to coronary heart disease (CHD) within 30 years as our binary response, bluntly ignoring censoring in order to keep the example simple and to obtain a relatively high prevalence, both of which render the presentation of the issues we would like to bring forward didactically easier. In what follows, only individuals for whom all variables were measured are analysed (*n *= 4658). Since we have a total of 1403 deaths within 30 years, our observed prevalence is *π *= 0.30.

We fitted two logistic regression models for the response variable: A simple model including only the explanatory variable serum cholesterol, and a complex model including the explanatory variables sex, age, BMI, serum cholesterol, diastolic blood pressure, and systolic blood pressure. For each of the two models, for the strategies to treat none or to treat all as well as for a hypothetical perfect prediction model, the net benefit was calculated and plotted against threshold probabilities *p_t _*ranging from 0 to 1 constituting five decision curves (Figure 1a). The complex model has a higher net benefit than the simple model over the whole range of *p_t_*, the difference being sizeable and visible on the graph for values of *p_t _*between c. 0.2 and 0.5 (Figure 1a). The two decision curves for the extreme strategies to treat none or all individuals serve as reference lines to judge whether a prediction model has any additional benefit. For the simple model, this is roughly the case for *p_t _*ranging between c. 0.15 and 0.35, and for the complex model, this is roughly the case for *p_t _*ranging between c. 0.15 and 0.5 (Figure 1a). The decision curve for a hypothetical perfect prediction model, which is equivalent to an omniscient strategy, serves as a reference line indicating the maximum net benefit that can be achieved (Figure 1a).

## The benefit for the untreated

However, there is no apparent reason to focus solely on the individuals who receive the treatment when calculating the net benefit. One could also be interested in the profit and loss for the individuals not receiving the treatment, i.e. for whom *p_i _*<*p_t_*. For these individuals, the expected profit will be *dL *and the expected loss will be *cP*, the resulting benefit being *U_untreated _*= *dL *- *cP*. We may note that, while *U_untreated _*will in general be different from *U_treated _*for a given prediction model, the difference between two models with respect to *U_untreated _*will be equal to the difference between two models with respect to *U_treated_*, that is,(2)

as can be deduced from Baker et al. [6]. Thus, a model maximising *U_treated _*at some threshold probability *p_t _*will also maximise *U_untreated _*at this threshold.

In the same spirit as Vickers et al. [1], who defined a net benefit for the treated, one could also define a net benefit for the untreated, dividing *U_untreated _*by *L* and yielding *u_untreated _*= *d *- *cP*/*L*, or, expressed as a function of *p_t_*,(3)

This is the formula that Vickers et al. [1] would have obtained if they had reversed the coding of the outcome (i.e. if they had considered the individuals with the disease as being those without the disease, and the treated as being the untreated). Importantly, while a model maximising *u_treated _*will also maximise *u_untreated_*, the difference between two models will (in general) be different with respect to *u_treated _*or *u_untreated_*, as we shall see in our example.

In the extreme case where all individuals are treated, one has *c *= *d *= 0 and *u_untreated _*= 0, whatever the threshold probability. In the other extreme case where none of the individuals is treated, one has *c *= *π*, *d *= 1 - *π *and *u_untreated _*= (1 - *π*) - *π*(1 - *p_t_*)/*p_t_*. This is an increasing function of the threshold probability, ranging from negative infinity up to one minus the prevalence. For a model achieving a perfect prediction, one has *b *= *c *= 0 and *u_untreated _*= 1 - *π*, whatever the threshold probability. For many prediction models, the empirical finding is that the net benefit for the untreated will be a roughly increasing function of the threshold probability, approximately ranging from 0 up to one minus the prevalence.

The net benefit for the untreated is a difference between two complementary proportions summing up to 1 - *π*, those profiting from not obtaining the treatment (since they really do not have the disease) minus those loosing by not obtaining the treatment (since they have the disease and would need the treatment), the latter weighted by *P*/*L*. Here too, the net benefit is equal to zero if the loss compensates the profit, and it can even be negative if the loss surpasses the profit. The maximum possible value of the net benefit for the untreated is 1 - *π*, one minus the prevalence of the disease. It is again only achieved by a perfect prediction model.

When the net benefit for the untreated is applied to the example introduced above, the zero reference line is now represented by the strategy of treating all individuals, while the strategy of treating none of the individuals is now represented by a monotonously increasing line (Figure 1b). The decision curve for a hypothetical perfect prediction model is in this example higher for the untreated than for the treated (because the prevalence is smaller than 0.5; Figure 1b). The net benefit for both the simple and the complex model surpasses the two extreme strategies in the same range of *p_t _*as in Figure 1a. Similarly, the complex model is superior to the simple model in the same range of *p_t _*as in Figure 1a. However, the difference between the two models with respect to *u_untreated _*is visible for values of *p_t _*between c. 0.1 and 0.5 (whereas it was visible only between c. 0.2 and 0.5 with respect to *u_treated_*). Thus, while the two models achieve roughly the same benefit with respect to *u_treated _*for a clinician using a threshold of say *p_t _*= 0.15, the complex model will appear to be superior to the simple model at that threshold with respect to *u_untreated_*. In this regard, the use of the net benefit for the treated or of the net benefit for the untreated is not equivalent when comparing different models.

## The overall benefit

If one wishes to define a measure of the net benefit for all individuals (the treated and the non-treated), it seems natural to add up the net benefits calculated for the treated and for the untreated, yielding an overall net benefit defined as *u_overall _*= (*a *+ *d*) - *bL*/*P *- *cP*/*L*, or, expressed as a function of *p_t_*,(4)

A model maximising *u_overall _*at some threshold probability *p_t _*will also maximise both *u_treated _*and *u_untreated _*at this threshold probability. On the other hand, the difference between two models with respect to *u_overall _*is equal to the sum of the differences between these models with respect to *u_treated _*and to *u_untreated_*, such that a visible difference between two models with respect to either of these quantities will also be visible with respect to *u_overall_*.

The overall net benefit can be interpreted as a difference of complementary proportions summing up to 1, those profiting from being correctly classified *a *+ *d *minus those loosing by being incorrectly classified *b *+ *c*, the latter appropriately weighted to account for the fact that the values of *P *and *L *are different. As for the net benefits for the treated and for the untreated, the overall net benefit is equal to zero if the loss compensates the profit and it can even be negative if the loss surpasses the profit. The maximum possible value of the overall net benefit is 1, which is achieved only for a perfect prediction model. This maximum value of 1 is certainly a sensible value since it implies that 100% of the individuals would profit from a diagnostic or prognostic test, which is precisely the case for a perfect prediction model.

Applying the concept of an overall net benefit to our example is equivalent to adding up the curves in Figure 1a and b, resulting in the curves in Figure 1c. The curves for the two extreme strategies of treating none and treating all are equal to the curves for the strategy of treating none in the case of the untreated and for the strategy of treating all in the case of the treated, respectively, since the complementary strategies are represented by zero lines (Figure 1c). The curves for the hypothetical perfect prediction model add up to 1 (Figure 1c). The curves for both the simple and the complex model now have the form of valley cross sections (Figure 1c). Again, the complex model surpasses the simple model over the whole range of *p_t_*, the difference between the two models with respect to *u_overall _*being clearly visible between c. 0.1 and 0.5.

## Relationships to ROC analysis

Vicker et al. [1] stress that with the introduction of decision curve analysis, they hope to overcome the schism between those solely interested in the accuracy and those solely interested in the utility of a prediction model. Whereas the latter select their threshold probability independently from the model as *p_t _*= *L*/(*L *+ *P*), which is a personal threshold as explained above, the former select their threshold probability (or cutoff probability, denoted here by *p_c_*) to maximise some well-defined criterion. Thus, the resulting optimal *p_c _*generally differs from model to model. There are many criteria which can be optimised, but as Baker et al. [6] point out, one of the most commonly applied ones is the Youden index, equal to sensitivity + specificity - 1. Baker et al. [6] show that the Youden index is equal to the so-called "science of the method", as again defined by Peirce [3], and as such does not bear the name of its actual author. Baker et al. [6] illustrate how these two kinds of threshold (the optimal *p_c _*or the personal *p_t_*) are related to a receiver operating characteristic (ROC) curve. 

**Figure 2:**
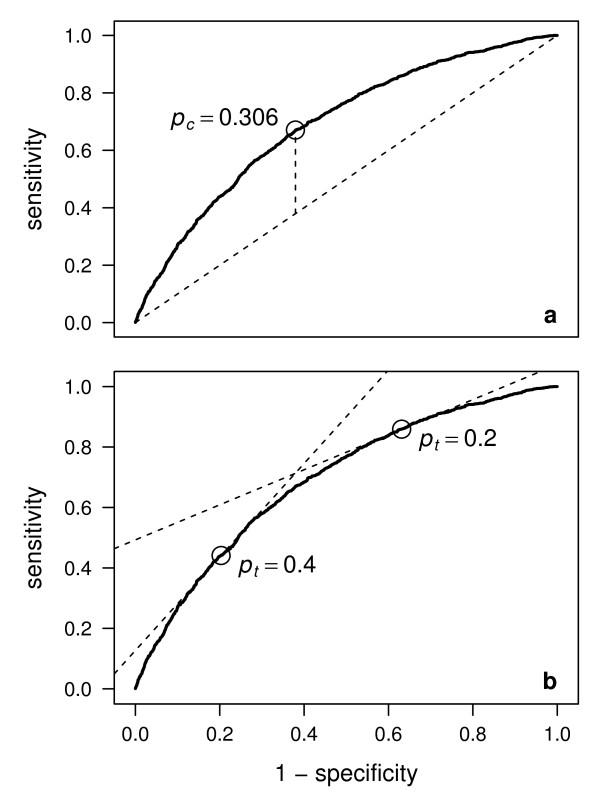
**Relationships of decision curve analysis to ROC curve analysis**. Receiver operating characteristic (ROC) curve for the complex example model. (a) The optimal operating point based on maximising the Youden index is the point on the curve with the largest vertical distance to the diagonal from the lower left corner to the upper right corner. The optimal operating point is indicated with a circle, the diagonal with a hatched line. (b) The optimal operating point based on maximising the net benefit is the point on the curve for which the slope is equal to [(1 - *π*)/*π*][*p_t_*/(1 - *p_t_*)]. Two examples for *p_t _*= 0.2 and *p_t _*= 0.4 are indicated with circles, the corresponding slopes with hatched lines.

Recall that a ROC curve shows all possible couples (1 - specificity; sensitivity) achievable by a prediction model when varying the threshold probability. The ROC curve of the complex model from our example is drawn in Figure 2. The threshold *p_c _*maximising the Youden index corresponds to the operating point on the ROC curve that is the highest point above the diagonal from the lower left to the upper right corner (Figure 2a). To determine the operating point on the ROC curve corresponding to the personal threshold *p_t _*= *L*/(*L *+ *P*), which maximises the personal benefit *u_treated _*= *a *- *bp_t_*/(1 - *p_t_*), first note that(5)

This follows from the fact that (estimates of) the sensitivity and the specificity are given by *a*/(*a *+ *c*) and by *d*/(*d *+ *b*), while (estimates of) *π *and 1 - *π *are given by *a *+ *c *and by *b *+ *d*. One may thus calculate the derivative of *u_treated _*with respect to 1 - specificity, given by(6)

and set it equal to zero, as explained in Baker et al. [6] (see also [7]). Thus *p_t _*corresponds to the operating point on the ROC curve for which the slope is equal to [(1 - *π*)/*π*][*p_t_*/(1 - *p_t_*)]. Steyerberg et al. [8] noticed that choosing *p_t _*= *π *implies *p_t _*= *p_c _*(as long as the optimal *p_c _*maximising the Youden index corresponds to the operating point on the ROC curve for which the slope is equal to 1). For our example, the solution for two selected values, *p_t _*= 0.2 and *p_t _*= 0.4, is given in Figure 2b.

**Figure 3:**
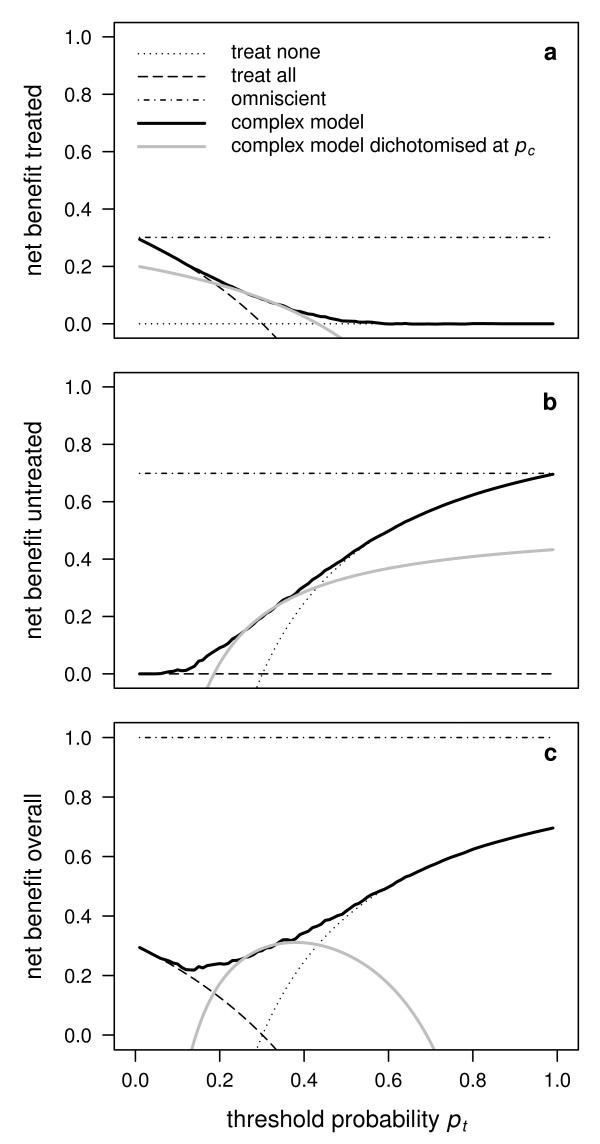
**Illustration of the net benefit achieved using an accurate binary diagnostic test**. Dichotomising the complex example model with the optimal ROC cutoff probability based on the maximum Youden index (or Peirce's "science of the method") results in an arc-shaped decision curve, with respect to the net benefit for the treated (a), the net benefit for the untreated (b), or the overall net benefit (c).

Whereas an ROC curve allows to demonstrate the sensitivity and the specificity achieved at each personal *p_t _*and to compare these values to what would be achieved using the optimal *p_c_*, it might also be of interest to show the net benefit achieved by the optimal *p_c _*as a function of *p_t_*. This is illustrated in Figure 3 for the complex model of our example. One can see that the net benefit quickly drops below the extreme strategies of treating none or all as soon as *p_t _*is departing from *p_c _*(which in our example was found to be 0.306). This underlines that the concepts of accuracy and utility of a prediction model may differ drastically in practice.

## Decision curve analysis in case-control studies

So far, we have implicitly assumed that our random sample was representative for the population of interest. If we now consider data from a case-control study, the empirical prevalence *π *will no longer be a consistent estimate of the true prevalence, which we shall denote by *π*_0_, since the cases are often overrepresented. In this context, it is well known that one can still use logistic regression to consistently estimate odds ratios. However, a *p_i _*calculated from a logistic regression model in a case-control study will no longer be a consistent estimate of the probability that the individual *i *has the disease. If one knew *π*_0_, this probability could be estimated by  defined such that(7)

that is(8)

To perform a decision curve analysis in a case-control study, one should hence know (or estimate) the true prevalence *π*_0 _using another source than the data themselves, typically from the literature. In this context, one may calculate  and opt for the treatment if and only if . Let then *a*' be the proportion of individuals with  and with the disease, let *b*' be the proportion of individuals with  and without the disease, let *c*' be the proportion of individuals with  and with the disease, and let *d*' be the proportion of individuals with  and without the disease (we further assume that an equality  is not possible, such that *a*' + *b*' + *c*' + *d*' = 1). In line with the fact that the empirical prevalence *π *= *a*' + *c*' is not a consistent estimate of the true prevalence *π*_0_, the quantities *a*', *b*', *c*' and *d*' are no consistent estimates of the true proportions of true positive, false positive, false negative and true negative results, respectively. Note however that *a*'/(*a*' + *c*') and *d*'/(*b*' + *d*') are still consistent estimates of the true sensitivity and the true specificity (recall also that an ROC analysis can still be carried out in a case-control study). To estimate the net benefit for the treated in a case-control study, one may thus use the mathematical expression given in the previous section, that is(9)(10)

Similarly, the net benefit for the untreated can be estimated as(11)(12)

and the overall net benefit as(13)

If the true prevalence is unknown, the net benefit is a function of both the personal threshold *p_t _*and the true prevalence *π*_0_. One can then perform several decision curve analyses over a plausible range of values of *π*_0_. Alternatively, for a given threshold *p_t_*, one could show the net benefit achievable by the prediction models under consideration in function of the unknown *π*_0_. Note that Pencina et al. [9] used a similar strategy to calculate a "net reclassification improvement" in the context of a case-control study.

**Figure 4:**
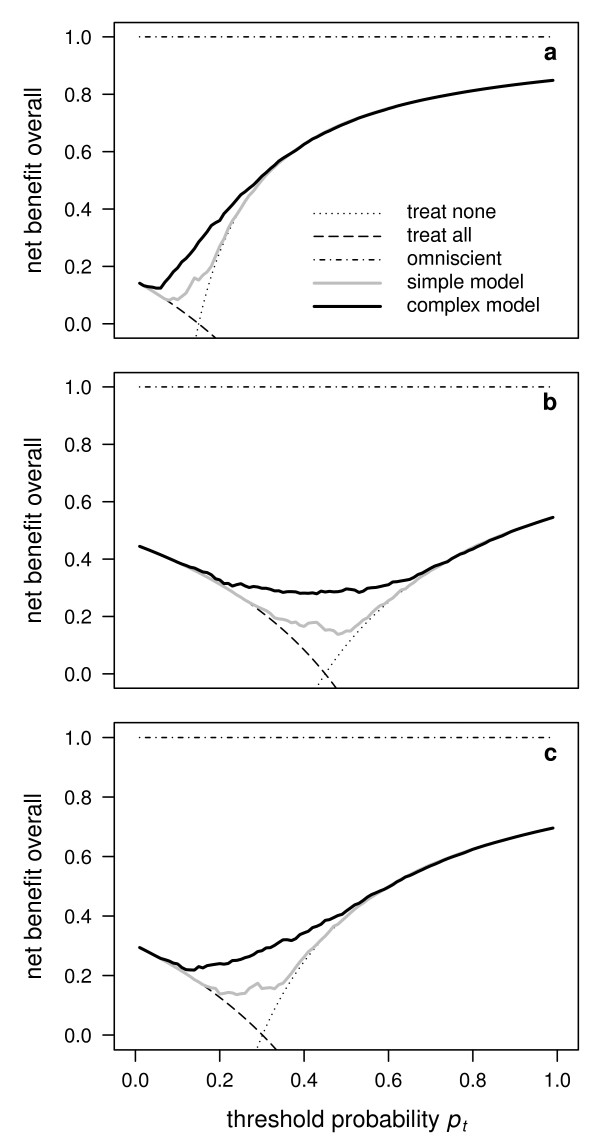
**Application of decision curve analysis to a case-control study**. Decision curves for a simple model, a complex model, the reference strategies to treat none or all, and a hypothetical perfect prediction model based on the overall net benefit for an assumed true prevalence of *π*_0 _= 0.15 (a), *π*_0 _= 0.45 (b) and *π*_0 _= 0.30 (c), the latter corresponding to the empirical prevalence *π*. While (c) is a reasonable assumption if the data are representative of the population of interest, this may no longer be the case if the data originate from a case-control study.

For illustrative purposes, let us pretend that the data of our example above originate from a case-control study and that the empirical prevalence *π *thus does not reflect the true prevalence *π*_0_. We have drawn three decision curves for which the true prevalence *π*_0 _is taken to be smaller, higher or equal to the empirical prevalence *π *(Figure 4). One can see that all decision curves are both shifted and lowered or raised depending on the value of *π*_0 _(Figure 4a, b and c). As a consequence, the range of *p_t _*where the prediction models have any additional benefit compared to the extreme strategies to treat none or all will depend on the postulated value of *π*_0_. More generally, a comparison among alternative prediction models (as well as the decision of which of them to select) will not only be made in function of the threshold probability *p_t _*but also of the assumed true prevalence *π*_0 _of the population of interest.

## Discussion

We have recalled the foundations of decision curve analysis and discussed some new aspects. In the original proposal, prediction models are compared with respect to the net benefit for the treated where one weighs the profits and losses for those receiving the treatment. Besides the net benefit for the treated, one could also be interested in the net benefit for the untreated which weighs the profits and losses for those not receiving the treatment, as developed above. In fact, the net benefit for the untreated would correspond to the net benefit for the treated if one would reverse the coding of the outcome. However, whereas a model maximising the net benefit for the treated will also maximise the net benefit for the untreated, the difference between two models will (in general) not be the same with respect to the net benefit for the treated or for the untreated. This actually implies that the original definition of the net benefit by Vickers et al. [1] is sensitive to the coding of the outcome, which may not be an optimal feature.

**Figure 5:**
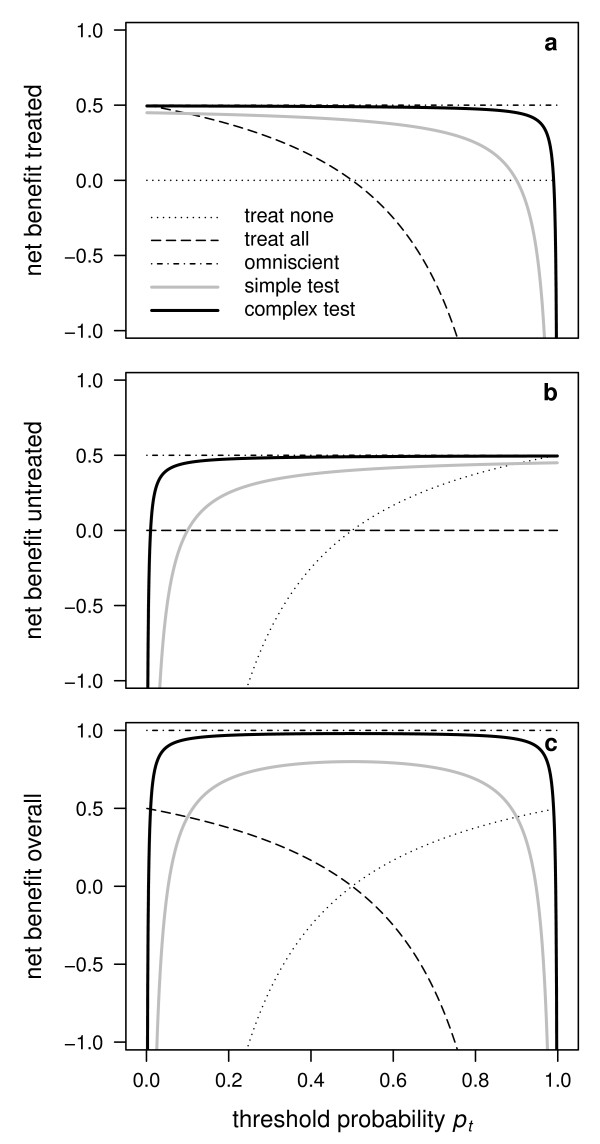
**Comparison of simple and complex binary diagnostic tests with respect to the net benefit**. At a low threshold probability *p_t_*, the difference between the two tests appears to be much higher with respect to the net benefit for the untreated (b) than with respect to the net benefit for the treated (a). The converse is true at a high probability threshold. A complete picture of the situation is conveyed using the overall net benefit (c). In this example, the simple test has 90% sensitivity and specificity and the complex test has 99% sensitivity and specificity in a context where the prevalence is 0.5.

To illustrate further how our clinical judgement could be altered when using one measure rather than the other when calculating the net benefit, we compare a simple binary diagnostic test achieving 90% of sensitivity and specificity with a complex binary diagnostic test achieving 99% of sensitivity and specificity in a context where the prevalence is 0.5 (Figure 5, a fictitious example). One can see that the difference between the two tests is rather small with respect to the net benefit for the treated at a low threshold value *p_t _*(Figure 5a), whereas it is much higher with respect to the net benefit for the untreated at the same threshold (Figure 5b). Thus, a clinician with a low personal threshold may conclude that the complex binary diagnostic test is not much more useful than the simple one from a clinical point of view if he/she looks at the net benefit for the treated, whereas his/her conclusion may drastically change if he/she looks at the net benefit for the untreated. Note that these differences are not a consequence of small sample problems and that the sample size appears in none of the formulae provided in this paper.

In order to define a measure of the net benefit which is invariant with respect to the coding of the outcome, one may combine the net benefits for the treated and the untreated in a symmetric way. In this paper, we have simply considered their sum, referred to as the "overall net benefit" (as done in Figure 5c of our fictitious example). Another interesting property of the overall net benefit is that the maximum possible value is always 1 (even though it is difficult to reach). In contrast, the maximum possible value for the net benefit proposed by Vickers et al. [1] is equal to the prevalence of the disease, which will be different from study to study. We also note that the overall net benefit is not more complicated to calculate than the net benefit for the treated. A user-friendly R package [10] to calculate and draw decision curves based on the overall net benefit is available from the second author upon request.

The crucial aspect of decision curve analysis is the choice of the threshold *p_t_*, which may be viewed as a potential limitation of the method since it can be difficult to quantify the consequences of a misclassification (the harms of being a false positive or a false negative). Fortunately, the choice of the threshold only depends on the relative expression *L*/*P*, not on the absolute terms *P *and *L*, which should already greatly simplify the practice. This was one motivation of Vickers at al. [1] to consider a "net benefit" (in our notation, *u_treated_*) rather than an "absolute benefit" (in our notation, *U_treated_*). Interpreting the value of a net benefit is however not quite straightforward and interpreting a difference between the net benefits achieved by two different models may still be more problematic. Recently, Tsalatsanis et al. [11] addressed this issue via a regret theory approach. On the other hand, the value of an absolute benefit would indeed be informative from a cost-effectiveness perspective (regarding the gain achieved when using the prediction model to decide whether an individual should be treated or not, compared to the strategy of treating nobody, as provided by *U_treated_*, compared to the strategy of treating everybody, as provided by *U_untreated_*, or compared to the strategy of doing the contrary of what is predicted by the model, as provided by the sum of *U_treated _*and *U_untreated_*).

We note that it might be difficult to define a measure of the utility of a model which is invariant with respect to the coding of the outcome, which only depends on the relative expression *L*/*P*, and which is easily interpretable. The overall net benefit considered in the present paper does satisfy the first two properties, while it may still be difficult to interpret. To assist interpretation, we described the overall net benefit as a difference of two complementary proportions. In the special case where the harms of a false positive equal the harms of a false negative (the threshold being *p_t _*= 0.5), the overall net benefit equals (*a *+ *d*) - (*b *+ *c*), which is simply the percentage of those correctly classified minus the percentage of those incorrectly classified. An overall net benefit of 0.2, for example, means that 60% of the subjects are correctly classified while 40% are not, the net benefit hence being 60% - 40% = 20%. In the more general case where different personal values are assigned to profits and losses, this difference of complementary proportions is weighted accordingly, such that a net benefit of 0.2 keeps the same "clinical value" in the context described above with *p_t _*= 0.5, or in another context.

In summary, we propose to use the overall net benefit instead of the benefit for the treated in a decision curve analysis when the issue is to assess and compare several concurrent prediction models with respect to their utility. We also took the opportunity to recall the important difference which may arise between the notions of accuracy and utility of a prediction model, as illustrated by Figure 3. Finally, we have illustrated how to use decision curve analysis in the context of case-control studies by allowing the prevalence, in addition to the threshold probability, to vary over a sensible range. We hope that our remarks will facilitate interpretation of decision curve analysis and motivate a more widespread adoption of the method.

## Abbreviations

*p_i_*: probability that an individual *i *has (or will develop) a disease of interest; *p_t_*: threshold probability above which one opts for treatment; *L*: loss caused by treating an individual without the disease of interest; *P *: profit obtained by treating an individual with the disease of interest; *a*: proportion of individuals with *p_i _*>*p_t _*with the disease of interest; *b*: proportion of individuals with *p_i _*>*p_t _*without the disease of interest; *c*: proportion of individuals with *p_i _*<*p_t _*with the disease of interest; *d*: proportion of individuals with *p_i _*<*p_t _*without disease of interest; *u_overall_*: overall net benefit; *U_treated_*: benefit for the treated; *u_treated_*: net benefit for the treated; *U_untreated_*: benefit for the untreated; *u_untreated_*: net benefit for the untreated; ROC: receiver operating characteristic; *π*: observed prevalence of the disease of interest; *π*_0_: true prevalence of the disease of interest.

## Competing interests

The authors declare that they have no competing interests.

## Authors' contributions

This paper is the result of numerous discussions between VR and TZ. Both authors participated actively in the writing and editing of the manuscript. Both authors read and approved the final draft.

## Pre-publication history

The pre-publication history for this paper can be accessed here:

http://www.biomedcentral.com/1472-6947/11/45/prepub

## References

[B1] Vickers A Elkin E Decision curve analysis: a novel method for evaluating prediction models Med Decis Making 2006 26 6 565 574 10.1177/0272989X06295361 17099194 PMC2577036

[B2] Fleiss J Levin B Paik M Statistical methods for rates and proportions 2003 3 Wiley New York

[B3] Peirce CS The numerical measure of the success of predictions Science 1884 4 453 454 17795531 10.1126/science.ns-4.93.453-a

[B4] Dupont W Statistical modeling for biomedical researchers: a simple introduction to the analysis of complex data 2002 2 Cambridge University Press

[B5] Levy D National Heart, Lung, and Blood Institute, Center for Bio-Medical Communication 50 years of discovery: medical milestones from the National Heart, Lung, and Blood Institute's Framingham Heart Study 1999 Hackensack, NJ: Center for Bio-Medical Communication

[B6] Baker SG Kramer BS Peirce, Youden, and receiver operating characteristic curves Am Stat 2007 61 4 343 346 10.1198/000313007X247643

[B7] Metz C Basic principles of ROC analysis Semin Nucl Med 1978 8 4 283 298 10.1016/S0001-2998(78)80014-2 112681

[B8] Steyerberg E Vickers A Cook N Gerds T Gonen M Obuchowski N Pencina M Kattan W Assessing the performance of prediction models -- a framework for traditional and novel measures Epidemiology 2010 21 128 138 10.1097/EDE.0b013e3181c30fb2 20010215 PMC3575184

[B9] Pencina M D'Agostino R Sr Steyerberg E Extensions of net reclassification improvement calculations to measure uefulness of new biomarkers Stat Med 2011 30 11 21 10.1002/sim.4085 21204120 PMC3341973

[B10] R Development Core Team R: A language and environment for statistical computing 2011 R Foundation for Statistical Computing, Vienna, Austria http://www.R-project.org

[B11] Tsalatsanis A Hozo I Vickers A Djulbegovic B A regret theory approach to decision curve analysis: a novel method for eliciting decision makers' preferences and decision-making BMC Med Inf Decis Making 2010 10 51 10.1186/1472-6947-10-51 PMC2954854 20846413

